# Functionally Distinct Circuits Are Linked by Heterocellular Electrical Synapses in the Thalamic Reticular Nucleus

**DOI:** 10.1523/ENEURO.0269-23.2023

**Published:** 2024-01-10

**Authors:** Mitchell J. Vaughn, Zachary Laswick, Huaixing Wang, Julie S. Haas

**Affiliations:** Department of Biological Sciences, Lehigh University, Bethlehem 18015, Pennsylvania

**Keywords:** electrical synapse, gap junction, thalamocortical, thalamus

## Abstract

The thalamic reticular nucleus (TRN) inhibits sensory thalamocortical relay neurons and is a key regulator of sensory attention as well as sleep and wake states. Recent developments have identified two distinct genetic subtypes of TRN neurons, calbindin-expressing (CB) and somatostatin-expressing (SOM) neurons. These subtypes differ in localization within the TRN, electrophysiological properties, and importantly, targeting of thalamocortical relay channels. CB neurons send inhibition to and receive excitation from first-order thalamic relay nuclei, while SOM neurons send inhibition to and receive excitation from higher-order thalamic areas. These differences create distinct channels of information flow. It is unknown whether TRN neurons form electrical synapses between SOM and CB neurons and consequently bridge first-order and higher-order thalamic channels. Here, we use GFP reporter mice to label and record from CB-expressing and SOM-expressing TRN neurons. We confirm that GFP expression properly differentiates TRN subtypes based on electrophysiological differences, and we identified electrical synapses between pairs of neurons with and without common GFP expression for both CB and SOM types. That is, electrical synapses link both within and across subtypes of neurons in the TRN, forming either homocellular or heterocellular synapses. Therefore, we conclude that electrical synapses within the TRN provide a substrate for functionally linking thalamocortical first-order and higher-order channels within the TRN.

## Significance Statement

The thalamic reticular nucleus (TRN) has key roles in sleep, attention, and seizures. Recent work has identified distinct primary and higher-order channels of thalamocortical information flow, but whether and how those channels interact within the TRN is unknown. Intra-TRN communication occurs via a dense network of electrical synapses. Here, we leveraged genetic tools and patch clamping to test whether electrical synapses couple TRN neurons of different subtypes and thereby provide a link between information channels. We found that TRN neurons promiscuously form electrical synapses within and across genetic subtypes, providing a new role for the electrical synapses of the TRN.

## Introduction

Arising sensory signals travel through the thalamus en route to further processing by the cortex. The thalamic reticular nucleus (TRN) is a shell of GABAergic neurons surrounding the dorsal thalamus (Pinault, 2004) that interface the flow of sensory signals by providing the major source of inhibition to thalamic relay neurons and nuclei (Minderhoud, 1971; Jones, 1975; Ohara, 1988; Pinault and Deschenes, 1998). The TRN receives inputs from most thalamic areas (Jones, 1975; Ohara and Lieberman, 1985; Fosse et al., 1986; Fitzpatrick et al., 1994; Liu and Jones, 1999) as well as deep layers of the cortex (Jones, 1975; Bromberg et al., 1981; Fonnum et al., 1981; Feig and Harting, 1998; Zhang and Jones, 2004; Whilden et al., 2021; Carroll et al., 2022) and a variety of modulatory sources (Anaya-Martinez et al., 2006; Zikopoulos and Barbas, 2012; Aizenberg et al., 2019). Inhibitory signals from the TRN to the thalamus are key regulators of sleep states (Steriade, 2005; Ni et al., 2016; Fernandez et al., 2018; Vantomme et al., 2019) and are responsible for thalamic and cortical spindle generation during sleep (Halassa et al., 2011; Thankachan et al., 2019) as well as cortical slow wave activity during sleep and absence seizures (Inoue et al., 1993; Destexhe, 1998; Steriade, 2005; Lewis et al., 2015; McCafferty et al., 2018). Due to its anatomical and circuit positioning, the TRN has been suggested to focus an attentional spotlight on specific items within the sensory surround (Crick, 1984; McAlonan et al., 2006), and several lines of behavioral evidence support its pivotal role in attention-based tasks (Zikopoulos and Barbas, 2012; Halassa et al., 2014; Wimmer et al., 2015; Sherman, 2016; Nakajima et al., 2019).

Within the TRN, subdivisions by primary sensory modalities have long been established (Pinault, 2004). Early work identified the TRN as a homogeneous population of GABAergic neurons that express parvalbumin (PV; Jones and Hendry, 1989; Seto-Ohshima et al., 1989; Frassoni et al., 1991) with some distinctions between cell types proposed based on cellular anatomy and molecular expression (Bendotti et al., 1990; Mitrofanis, 1992b; Cox et al., 1996; Crabtree, 1996; Lizier et al., 1997; Pinault and Deschenes, 1998; Lam and Sherman, 2007). Recent results leveraging genetic targeting have established a more precise subdivision of TRN neurons, which ties molecular expression to thalamic connectivity. These were first distinguished by PV and somatostatin (SOM) expression (Clemente-Perez et al., 2017). Subsequent work has also distinguished types by calbindin (CB) and SOM expression (Martinez-Garcia et al., 2020) and by a gradient of transcription factor expression (Li et al., 2020). SOM neurons expressing the transcription factor Ecel1 compose the outer shell of the TRN, while PV or CB neurons expressing the transcription factor SPP1 compose the core of the TRN. These subtypes also differ in intrinsic neuronal properties, such as the low-threshold calcium current underlying bursts of action potentials (Clemente-Perez et al., 2017; Li et al., 2020; Martinez-Garcia et al., 2020). Importantly, these distinct neuronal subtypes have also been shown to reciprocally synapse with functionally distinct thalamic targets: SOM neurons reciprocally connect to higher-order thalamic nuclei, areas driven by inputs from cortex, while CB or PV neurons reciprocally connect to the first-order thalamus, areas that are driven by prethalamic afferents (Clemente-Perez et al., 2017; Martinez-Garcia et al., 2020). Cortical input from layers 5 and 6 to the TRN is also organized by shell and core distributions (Whilden et al., 2021; Carroll et al., 2022). Together, the first-order thalamocortical channel consists of primary thalamic nuclei, CB TRN neurons, and cortical input from layer VI, while the higher-order thalamocortical channel comprises higher-order thalamic nuclei, SOM TRN neurons, and cortical input from both layers V and VI. Whether and how these parallel information channels interact within the TRN is unknown.

Synaptic connections between TRN neurons may include GABAergic contacts (Deleuze and Huguenard, 2006; Lam and Sherman, 2007), although these seem to be pruned by the second postnatal week (Hou et al., 2016). The main source of connectivity within the TRN is thought to be its dense and powerful electrical synapses (Landisman et al., 2002; Hou et al., 2016). These synapses have been shown to coordinate TRN rhythms (Fuentealba et al., 2004; Long et al., 2004; Haas and Landisman, 2011), temporally shape the relay of signals through thalamocortical channels (Pham and Haas, 2018), and depress or potentiate as a result of the two distinct TRN activity patterns, bursting, and tonic spikes (Haas et al., 2011; Fricker et al., 2021). Electrical synapses are stronger and more common with closer intersomatic distance (Landisman et al., 2002; Deleuze and Huguenard, 2006; Lee et al., 2014). Understanding of the organization of electrical synapses and networks within the TRN is thus far limited to anatomical work, where dye coupling has shown that network shape appears to vary with position (Lee et al., 2014). Whether electrical synapses couple similar or disparate sets of TRN neurons is unknown. Across the central nervous system, electrical synapses have mostly been shown to couple similar classes or subtypes of neurons (Galarreta and Hestrin, 1999; Gibson et al., 1999; Onn and Grace, 1999; Tamás et al., 2000; Venance et al., 2000; Deans et al., 2001; Schoppa and Westbrook, 2002; Beierlein et al., 2003; Blatow et al., 2003; Chu et al., 2003; Galarreta et al., 2004; Dugué et al., 2009; Curti et al., 2012; Fernandez et al., 2022), but reports of coupling between electrophysiologically distinct classes have been made (Venance et al., 2000; Simon et al., 2005; Zsiros and Maccaferri, 2005; Bloomfield and Volgyi, 2009; Apostolides and Trussell, 2013; Hatch et al., 2017; Kocaturk et al., 2022). In the retina, coupling between cone bipolar cells mixes signals from otherwise parallel channels (Sigulinsky et al., 2020).

To determine whether thalamocortical information channels interact via electrical synapses in the TRN, we used SOM-Cre and CB-Cre mice to identify and electrophysiologically characterize a large cohort of TRN neurons of each genetic subtype in vitro. Our results describe a set of intrinsic passive and active differences between genetically labeled SOM and CB neurons. We note that acceleration within a burst, a criterion often used to identify TRN neurons in recordings in vivo, is robust for CB neurons but rare for SOM neurons. Our recordings revealed electrical synapses linking both TRN neurons of the same subtype (homocellular synapses) and different subtypes (heterocellular synapses). Heterocellular coupling was confirmed by differences in electrophysiological properties between coupled neurons that matched their genetic labeling. We conclude that heterocellular electrical synapses in the TRN form networks that provide a basis for synaptic interactions between thalamocortical first-order and higher-order channels.

## STAR Methods

### Contact for reagent and resource sharing

Further information and requests for resources and reagents should be directed to and will be fulfilled by the lead contact.

### Experimental model and subject details

All experiments were performed in accordance with the federal IACUC animal welfare guidelines.

#### Animals

We used offspring from SOM-Cre (Jax: 013044) and CB-Cre mice (Jax: 028532) crossed with Ai6 GFP reporter mice (Jax: 007906) of both sexes aged postnatal days (P) 11–28. Mice were anesthetized by inhaled isoflurane (5 ml of isoflurane applied to fabric, within a 1 L chamber) and killed via decapitation.

#### Electrophysiology

Horizontal brain slices 300 µm thick were cut and incubated in sucrose solution (in mM): 72 sucrose, 83 NaCl, 2.5 KCl, 1 NaPO_4_, 3.3 MgSO_4_, 26.2 NaHCO_3_, 22 dextrose, 0.5 CaCl_2_. Slices were incubated at 37°C for 30 min in sucrose following cutting and returned to ACSF at room temperature until recording. The artificial cerebral spinal fluid bath during recording contained the following (in mM): 126 NaCl, 3 KCl, 1.25 NaH_2_PO_4_, 2 MgSO_4_, 26 NaHCO_3_, 10 dextrose, and 2 CaCl_2_, 315–320 mOsm L^−1^, saturated with 95% O_2_/5% CO_2_. The submersion recording chamber was held at 34°C (TC-324B, Warner Instruments). Electrodes were ﬁlled with the following (in mM): 135 potassium gluconate, 2 KCl, 4 NaCl, 10 Hepes, 0.2 EGTA, 4 ATP-Mg, 0.3 GTP-Tris, and 10 phosphocreatine-Tris, pH 7.25 (295 mOsm L^−1^). A 1 M KOH was used to adjust pH of the internal solution. The approximate bath ﬂow rate was 2 ml min^−1^, and the recording chamber held ∼5 ml solution.

The TRN was visualized under 4× magniﬁcation, and pairs of adjacent TRN cells from any sensory sector were identiﬁed and patched under 40× IR-DIC optics (SliceScope, Scientiﬁca). Cell type was initially determined by GFP epifluorescence in the patched neurons. Cells with low fluorescent contrast were avoided. The GFP reporter was excited by a 472 nm diode delivered through the objective (CoolLED pE-300). Voltage signals were ampliﬁed and low-pass ﬁltered at 8 kHz (MultiClamp, Axon Instruments, Molecular Devices) and digitized at 20 kHz with custom Matlab routines controlling a National Instruments (USB6221 DAQ board), and data were stored for ofﬂine analysis in Matlab (MathWorks, R2018b). Recordings were made in whole-cell current-clamp mode. Negative current was used to maintain cells at −70 mV during recordings. Current injections (500 ms) were used to measure intrinsic properties and coupling. Pipette resistances were 5–9 MΩ before bridge balance; recordings were discarded if access resistance exceeded 25 MΩ. Voltages are reported uncorrected for the liquid junction potential.

#### Numerical analysis

Significance tests are two-tailed Mann–Whitney. To measure intrinsic electrophysiological properties, we stimulated each neuron of a pair separately with 500 ms current pulses from −100 to +250 pA in steps of 25 pA. *R*_in_ was calculated using the change in membrane voltage divided by the change in applied current, averaged across the first four stimuli (−100, −75, −50, −25 pA). Peak burst rate was the inverse of the fastest interspike interval during a burst, computed for the first trace in which the neuron spiked (rheobase). We identified bursts as the spikes within the first 100 ms after the first spike, or before firing rate fell below 20 Hz, whichever came first. FI gain for tonic spikes was computed as the average firing rate in the last 200 ms of stimulation divided by the amplitude of the applied current, averaged over the of the set of traces in which the neuron spiked during that time. Acceleration was measured by dividing the first interspike interval by the smallest interspike interval, computed for the rheobase burst and bursts with >2 spikes. Instantaneous frequency was the inverse of each interspike interval. We measured coupling between neurons by delivering a −100 pA, 500 ms current to one neuron and measuring voltage deflections in both neurons. The amplitude of hyperpolarizing current was chosen to minimize activation of voltage-dependent conductances that could distort coupling measurement (Curti and Pereda, 2004; Haas and Landisman, 2011; Li et al., 2023). These currents were repeated 10 times for each neuron of the pair. Coupling coefficient (cc) was calculated as the ratio of voltage changes, of unstimulated neuron to the stimulated neuron, averaged over 10 trials and averaged over both directions of measurement. Coupling conductance was estimated as the inverse of junctional resistance (Fortier, 2010; Haas et al., 2011).

#### Data availability

Datasets collected and analyzed from this study are available upon reasonable request to the corresponding author.

## Results

### SOM and CB neurons are distinguished by differences in intrinsic and bursting properties

SOM and CB (or PV) neurons have distinguishable electrophysiological properties in adult tissue, particularly differences in bursting properties (Clemente-Perez et al., 2017; Li et al., 2020; Martinez-Garcia et al., 2020). All previous electrophysiological investigations of electrical synapses in the TRN using paired recordings have used juvenile tissue (Landisman et al., 2002; Landisman and Connors, 2005; Parker et al., 2009; Haas et al., 2011; Haas and Landisman, 2011; Oh et al., 2014; Sevetson and Haas, 2015; Wang et al., 2015; Kohmann et al., 2016; Zolnik and Connors, 2016; Sevetson et al., 2017; Fricker et al., 2021) due to the visual occlusion that results from relay neuron myelination in adult tissue. Our goal to widely sample coupling between TRN neuronal subtypes was subject to the same limitation. Juvenile TRN neurons of the age range used here have acquired adult properties (Warren and Jones, 1997; Degen et al., 2004; Cueni et al., 2008; Parker et al., 2009). We ultimately aimed to test for coupling between neurons of different subtypes labeled by GFP in reporter-crossed mice. A possible confound for our coupling measurements is overexpression of GFP due to genetic recombination early in development that might result in persistent GFP reporting (Hu et al., 2013) in crossed mice, which could blur distinctions between cell types. Thus, we first aimed to confirm that our GFP-identified juvenile SOM and CB neurons exhibit distinct sets of intrinsic and spiking properties that have been distinguished in adulthood.

**Figure 1.**
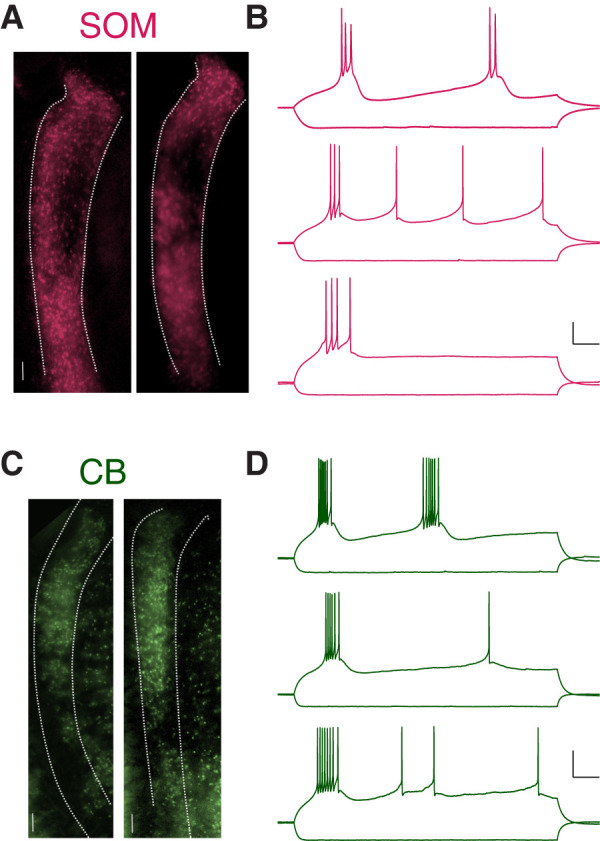
Example spatial distributions and spiking responses of SOM and CB neurons in the TRN. ***A***, Fluorescence images of a live slice from a SOM_ _×_ _Ai6 mouse. SOM-GFP expression, pseudocolored in magenta here, occupies the shell of the TRN. Boundaries were drawn from corresponding IR image. ***B***, Example responses of SOM neuron to current steps of −100 pA and rheobase for each cell. From bottom to top, *V*_m _= −74.5, −74.0, −77.0 mV. Scale bars, 20 mV, 50 ms. ***C***, CB-GFP expression in the TRN. ***D***, Example responses of CB neuron to current steps of −100 pA and rheobase for each cell. From bottom to top, *V*_m _= −74.9, −72.7, −77.3 mV. Scale bars, 20 mV, 50 ms. See Extended Data Figure 1-1 for an example of a recording at the boundary between the core and shell of the TRN.

We crossed SOM-Cre (Jax: 013044) or CB-Cre mice (Jax: 028532) with Ai6 GFP reporter mice (Jax: 007906) in order to label specific TRN neuronal subtypes with GFP. We denote neurons that do and do not express GFP in SOM-Cre × GFP mice as SOM and non-SOM, respectively, and similarly CB or non-CB for progeny of CB-Cre mice crossed with the Ai6 GFP reporter. In live fluorescence and differential interference contrast (DIC) images of horizontal slices containing the TRN prepared from progeny aged P11-P28, SOM neurons were distributed along the edges of the TRN (Fig. 1*A*) and CB neurons were densest in the core (Fig. 1*C*), similar to the previous report (Martinez-Garcia et al., 2020) of virally injected animals.

10.1523/ENEURO.0269-23.2023.f1-1 Figure 1-1 **A.** Example of within-TRN location of boundaries between expressing and non-expressing areas targeted for paired recordings Left: IR image. Right: GFP image. **B**. Pair targeted in ATRN. FO, first order; HO, higher order. Download Figure 1-1, TIF file.

We recorded from a total of 154 SOM and 107 CB TRN neurons identified by GFP expression, as well as 53 non-GFP-expressing neighboring cells, in current clamp from juvenile brain slices containing the TRN. We maintained neurons at −70 mV in current clamp and applied current steps ranging from −100 to +250 pA in steps of 25 pA to each neuron; the sub- and supra-threshold responses (Fig. 1*B*,*D*) to current steps allowed us to measure a host of electrophysiological properties and to test for coupling between those pairs. All neurons recorded were targeted for paired recordings and were in close proximity (measured between soma centers: 16.5 ± 0.9 µm in SOM mice and 17.9 ± 1.1 µm in CB mice). While we did not target specific sectors of the TRN for recording, most recordings of neurons with mismatched expression were made at expression boundaries, where GFP expression was most mosaic (Extended Data Fig. 1-1). Consistent with previous reports from adult tissue, we noted that juvenile SOM neurons appeared to have larger input resistance, and their bursts had fewer, slower spikes (Fig. 1*B*). Spikes within a burst from CB neurons (Fig. 1*D*) were faster and more numerous. Both SOM and CB neurons emitted tonic spikes following bursts (Fig. 1*B,D*).

**Figure 2.**
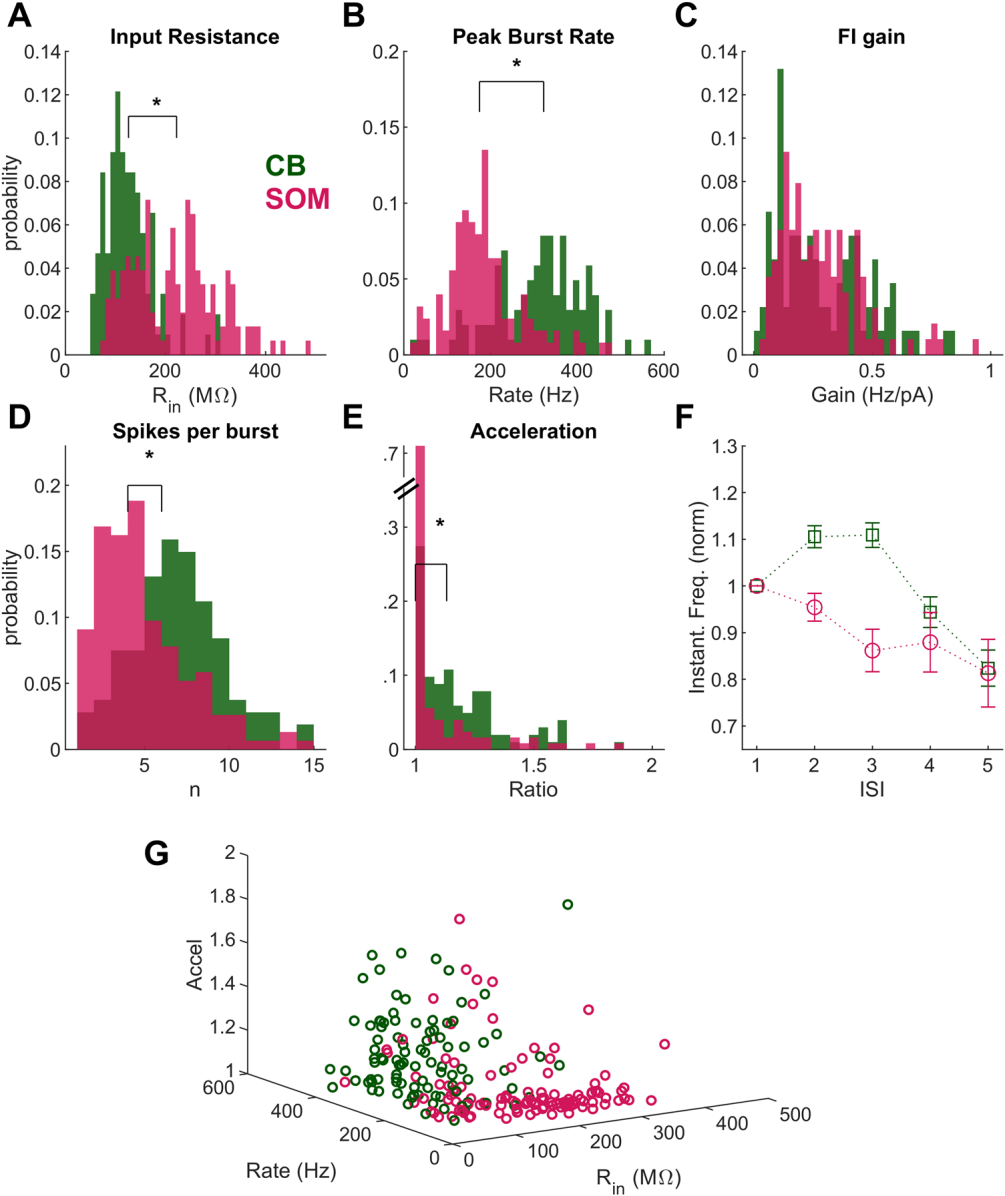
Intrinsic and bursting properties distinguish GFP + SOM and CB subtypes in the TRN. ***A***, *R*_in_ for CB (mean, 124.6 ± 4.7 MΩ) and SOM (222.3 ± 7.7 MΩ; *p* < 0.01; *n* = 107 CB and 154 SOM) neurons. ***B***, Peak instantaneous firing rate within bursts for SOM (mean, 184.4 ± 7.5 Hz) and CB (312.5 ± 10.6 Hz; *p* < 0.01) neurons. ***C***, Gain of tonic spiking frequency for SOM and CB neurons (SOM, 0.37 ± 0.03 Hz/pA; CB, 0.30 ± 0.02 Hz/pA; *p* = 0.5). ***D***, CB neurons fired more spikes for each burst (SOM, 4.8 ± 0.3 spikes; CB, 7.0 ± 0.3 spikes; *p* < 0.01). ***E***, Spiking within a burst accelerated (expressed as the ratio of fastest to first instantaneous rate within the burst) for CB neurons more than for SOM neurons (SOM: median, 1.0, mean, 1.1 ± 0.03; CB: median, 1.13, mean, 1.20 ± 0.02; *p* < 0.01). ***F***, Instantaneous firing for each interspike interval (ISI) within bursts, normalized to the first interval. Data are mean ± SEM. ***G***, Cell identity as a function of peak burst rate, input resistance, and acceleration ratio. See Extended Data Figure 2-1 for input resistance across age.

In order to fully characterize differences between GFP-identified SOM and CB neurons, we measured and compared distributions of input resistance, resting membrane potential, threshold, peak spiking rate within a burst, tonic firing gain, maximum tonic spiking rate, acceleration of spikes within a burst, and spikes per burst. In our dataset and similar to a previous report for young adult TRN (Martinez-Garcia et al., 2020), SOM neurons had substantially higher input resistance than CB neurons (Fig. 2*A*; mean CB *R*_in_ 124.6 ± 4.7 MΩ; mean SOM *R*_in_ 222.3 ± 7.7 MΩ; *p* < 0.01; Mann–Whitney test). Consistent with the report that *R*_in_ has stabilized by P11 to ∼200 MΩ (Zolnik and Connors, 2016), *R*_in_ did not change over the age range of our dataset (Extended Data Fig. 2-1). We found that SOM neurons were slightly depolarized relative to CB neurons (*V*_m_, mean CB −70.0 ± 0.6 mV; mean SOM −66.6 ± 0.8 mV; *p* < 0.01).

10.1523/ENEURO.0269-23.2023.f2-1 Figure 2-1 Input resistance (mean ± SEM) over postnatal age for the dataset used. Fits (dotted lines) are linear. Download Figure 2-1, TIF file.

Like most thalamic neurons (Steriade and Llinás, 1988; Contreras et al., 1992), TRN neurons fire action potentials in both a calcium burst crowned by a fast barrage of sodium spikes and in trains of regular, or tonic, spikes. We used the burst driven by the rheobase current for each neuron to compare bursts between GFP-expressing types. Several aspects of bursts varied between our SOM and CB TRN neurons. Similar to previous reports, SOM neurons fired fewer spikes within each burst than CB neurons (Fig. 2*D*; SOM, 4.8 ± 0.3 spikes; CB, 7.0 ± 0.3 spikes; *p* < 0.01). Nineteen SOM neurons and five CB neurons fired only one spike per burst. Within bursts, peak frequency (Fig. 2*B*) for SOM neurons (184.4 ± 7.5 Hz) was substantially slower than that for CB neurons (312.5 ± 10.6 Hz; *p* < 0.01). Spike threshold was lower for SOM neurons (threshold: mean CB, −40.1 ± 0.6 mV; mean SOM, −45.2 ± 1.1 mV; *p* = 0.01). Gain of tonic spiking did not differ between CB and SOM neurons (Fig. 2*C*; mean for SOM, 0.37 ± 0.03 Hz/pA; CB, 0.30 ± 0.02 Hz/pA; *p* = 0.5).

Many reports identify TRN neurons as accelerating within a burst; this is a criterion that is often used to identify TRN neurons within in vivo recordings. We measured instantaneous frequency within a burst and quantified acceleration as the ratio of the fastest frequency to the frequency of the first interspike interval within the burst for bursts with >2 spikes (Fig. 2*D*). In SOM neurons, this ratio was most frequently 1 (median, 1.0; mean, 1.1 ± 0.03; *n* = 126 neurons), indicating a lack of acceleration within bursts. In CB neurons, the ratio was significantly larger (median, 1.13; mean, 1.20 ± 0.02; *p* < 0.01; *n* = 102 neurons for comparison with SOM), reflecting the acceleration commonly observed. Normalized instantaneous frequencies within bursts are shown in Figure 2*E*, which confirms that the pattern of accelerating spikes within a burst distinguishes CB neurons from SOM neurons in the TRN. Together, our results show that spiking during bursts but not tonic spikes are a distinguishing feature for TRN neurons.

Collectively, these results confirm previous reports that TRN neurons take on mature intrinsic and spiking characteristics by the end of the second postnatal week. Further, they establish that juvenile TRN neurons have already differentiated into the identified SOM and CB subtypes observed in adults and that we can successfully distinguish between those neurons via a combination of GFP labeling and electrophysiological properties (Fig. 2*G*).

### SOM and CB neurons in the TRN are coupled by both homocellular and heterocellular electrical synapses

Having established that neurons in our juvenile tissue from crossed reporter mice are distinguishable by the same intrinsic and spiking properties that distinguish those neuronal subtypes in adult tissue, we set out to test whether the populations of neuronal subtypes defined by those properties form homocellular and heterocellular electrical synapses. Since it has been previously established that SOM and CB subtypes each interact with distinct thalamocortical information channels, identification of electrical synapses between subtypes will also determine possible avenues for cross talk between those channels within the TRN.

**Figure 3.**
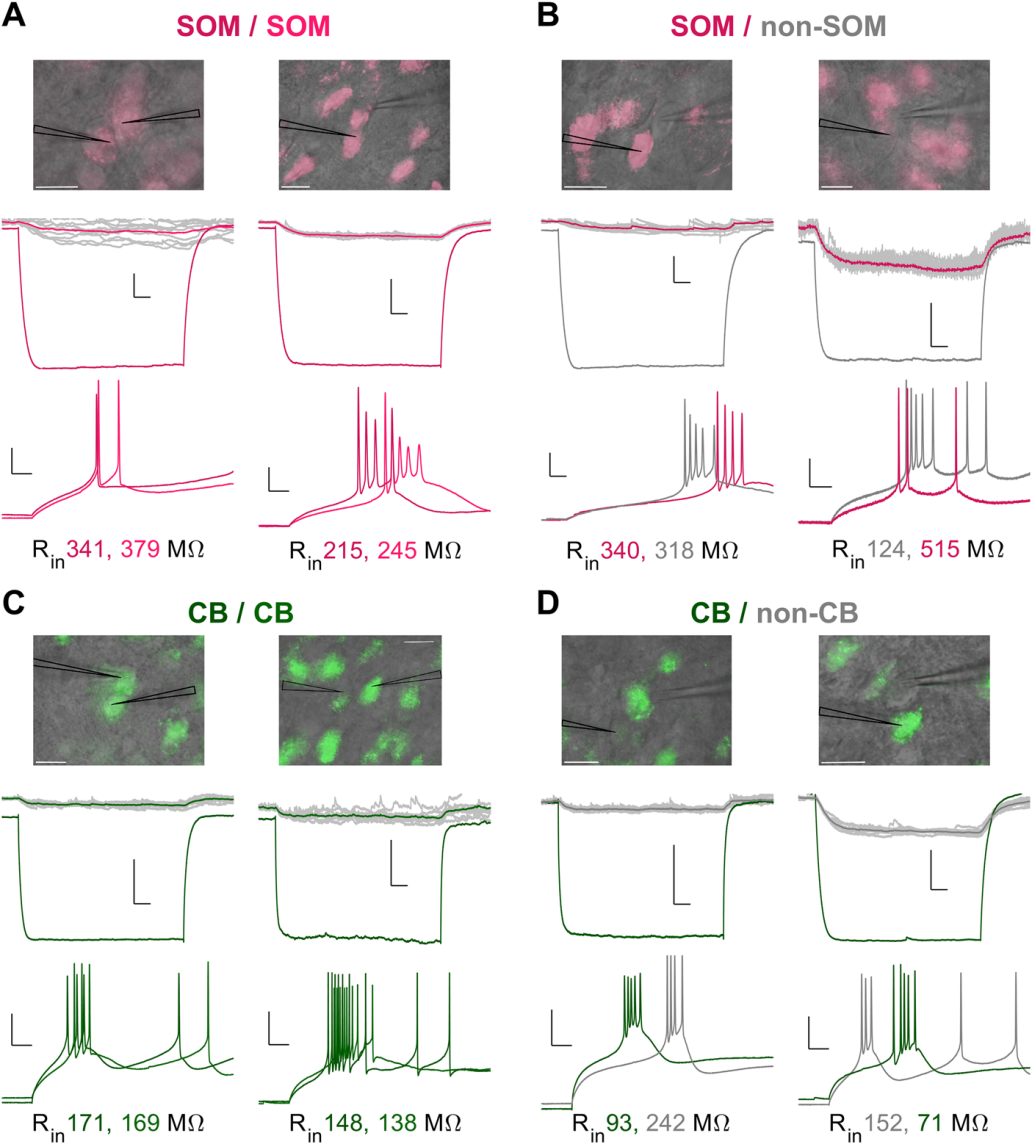
Homocellular and heterocellular coupling between pairs of TRN neurons. ***A***, Example of coupling between two pairs of SOM neurons. Top, Overlay of fluorescence and DIC images taken during live recordings. Scale bar, 20 µm. Middle, Simultaneous voltage responses of both neurons to −100 pA current steps delivered to one neuron of each pair. Scale bars, 5 mV, 50 ms. Bottom, Spiking responses of each neuron to separate injections of rheobase current. Scale bars, 20 ms, 20 mV. ***B***, As for ***A***, but for pairs of SOM and non-SOM neurons; scale bars in top right are 2.5 mV, 50 ms. ***C***, As for ***A***, but for pairs of CB neurons. ***D***, As for ***A***, but for pairs of CB and non-CB neurons.

We tested for the presence of homocellular or heterocellular electrical synapses between TRN neurons by patching adjacent pairs that were either SOM/SOM (*n* = 60 pairs), SOM/non-SOM (*n* = 34 pairs), CB/CB (*n* = 44 pairs), or CB/non-CB (*n* = 19 pairs), as defined by GFP expression. These pairs were composed of the same neurons used to evaluate electrophysiological properties. We tested each pair for the presence of an electrical synapse by applying a 100 pA hyperpolarizing current to one neuron and measuring a hyperpolarizing deflection in the coupled neighbor (Fig. 3*A*, middle traces). This test was repeated in both directions of current flow across the synapse. Examples of coupling measurements are shown in Figure 3 for SOM/SOM pairs (Fig. 3*A*), SOM/non-SOM pairs (Fig. 3*B*), CB/CB pairs (Fig. 3*C*), and CB/non-CB pairs (Fig. 3*D*). We identified electrical synapses between all combinations of paired subtypes patched.

**Figure 4.**
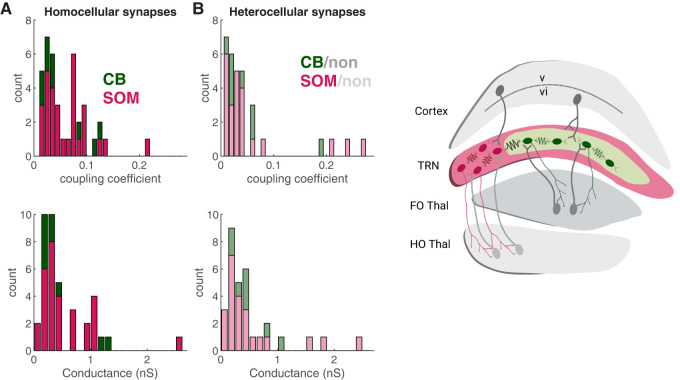
Distribution of coupling within and without SOM and CB cell types. ***A***, Stacked distribution of homocellular electrical synapses in the TRN; coupling was found between 9 out of 44 pairs of CB neurons tested and 30 out of 60 pairs of SOM neurons tested. cc, coupling coefficient. ***B***, Stacked distribution of heterocellular electrical coupling between TRN neurons: synapses were identified between 8 out of 19 pairs of CB and non-CB neurons and 23 out of 34 pairs of SOM and non-SOM neurons tested. ***C***, Schematic of distinct thalamocortical pathways linked by electrical synapses of the TRN. FO, first order; HO, higher order. See Extended Data Figure 4-1 for signal-to-noise of coupling measurements.

For coupled pairs, we calculated coupling coefficient (cc) as the voltage deflection of the unstimulated neuron divided by the voltage deflection in the neuron that received current steps. We considered coupling to exist for cc > 0.01, based on detectable voltage signals (Extended Data Fig. 4-1). We tested for coupling between a total of 157 pairs that included at least one GFP + neuron. Of our 157 total pairs tested, we found coupling between 30 homocellular SOM/SOM pairs (mean cc = 0.06 ± 0.008; mean *G*_C _= 0.30 ± 0.11 nS) out of 60 tested pairs and nine homocellular CB/CB pairs (mean cc = 0.05 ± 0.014; mean *G*_C _= 0.39 ± 0.14 nS) out of 44 tested pairs (Fig. 4*A*). We found coupling between 23 heterocellular SOM/non-SOM pairs (mean cc = 0.06 ± 0.016; mean *G*_C _= 0.25 ± 0.15 nS) out of 34 tested pairs, and eight heterocellular CB/non-CB pairs (mean cc = 0.05 ± 0.021; mean *G*_C _= 0.32 ± 0.14 nS) out of 19 tested pairs (Fig. 4*B*). These counts reflect a coupling frequency of 50% for SOM pairs and 21% for CB pairs. There were no significant differences between coupling coefficients or conductances between any cell or synapse types, and the frequency of coupling for CB/non-CB and SOM/non-SOM was not significantly different (*p* = 0.09; Fisher's test).

10.1523/ENEURO.0269-23.2023.f4-1 Figure 4-1 Signal-to-noise of coupling measurements. SNR is change in membrane voltage in the receiving neuron during repeated current steps (see Methods) divided by the standard deviation of voltage. Heterotypic pairs are blue, homotypic pairs are in red; circles represent SOM data, and squares represent CB data. Line at cc = 0.01 represents threshold used to identify electrical coupling. Download Figure 4-1, TIF file.

Our data lead us to conclude that unlike interneurons of cortex that preferably form homocellular electrical synapses (Gibson et al., 1999; Beierlein et al., 2003; Blatow et al., 2003; Chu et al., 2003; Galarreta et al., 2004), TRN neurons frequently form both homocellular and heterocellular electrical synapses between neurons of both similar and different types. We suggest that these synapses provide an avenue for interactions between first-order and higher-order thalamocortical relay channels (Fig. 4*C*).

## Discussion

Cortical attention to the sensory environment is regulated and focused by inhibition from the TRN, an area that also provides one possible key locus for mixing of cortex-bound sensory information. Here, we demonstrate that TRN neurons develop into mature and distinguishable SOM and CB subtypes in juvenile animals, prior to myelination of thalamocortical fibers. Further, we show that SOM and CB neurons form both homocellular and heterocellular electrical synapses. Because SOM neurons have reciprocal connections with higher-order thalamus while CB/PV neurons have distinct reciprocal connections to first-order thalamus (Clemente-Perez et al., 2017; Martinez-Garcia et al., 2020; Hoseini et al., 2021), and given that the two cell types receive inputs from distinct deep layers of cortex (Whilden et al., 2021; Carroll et al., 2022), our results imply that heterocellular coupling links the otherwise distinct first-order and higher-order thalamocortical channels. Conversely, homocellular coupling processes information sent within a thalamocortical relay channel.

We hypothesize that the functions of homocellular and heterocellular synapses share a function of modulating the inhibition delivered to the thalamus, but the impacts of that modulation vary for each synapse type. Coupled TRN neurons of similar subtype could be expected to have similar (but not identical) inputs and outputs, causing homocellular synapses to enhance TRN responses and sensitivity to shared inputs within a sensory modality, resulting in increased and/or better-coordinated thalamic inhibition as a result. Heterocellular coupling provides a substrate for higher-order processes to influence first-order thalamocortical sensory response and relay, dampening it through shared depolarization within the TRN that results in more inhibition to first-order thalamus. Conversely, heterocellular coupling could enhance thalamocortical sensory throughput via shunting across TRN electrical synapses that results in reduced inhibition in the thalamus. Electrical synapses generally facilitate synchronous firing between coupled neurons when they are simultaneously and persistently depolarized (Galarreta and Hestrin, 1999; Gibson et al., 1999; Landisman et al., 2002) and are thus proposed to support rhythms of coupled networks; the variety of coupling shown here could support trans-TRN synchrony initiated by one neuronal subtype.

For transient signals representing sensory inputs to the thalamus, electrical synapses have myriad and more complex effects (Connors, 2017; Alcami, 2018; Vaughn and Haas, 2022). Our previous modeling suggests that TRN electrical synapses aid input discrimination by modulating spike timing in relay neurons (Pham and Haas, 2018), and this effect could be implemented by homocellular synapses to discriminate between similar inputs of the same sensory modality. We noted that coupling was more prevalent between and from SOM neurons, implying that information mixing through electrical synapses is more influential in higher-order thalamocortical systems compared with primary sensory relay.

The heterotypic electrical synapses in our data had mean ccs near 0.05. The strength of synapses we show herein is within the range we previously showed through computational models to alter thalamocortical relay (Pham and Haas, 2018) and coordination of TRN spiking (Mendoza and Haas, 2022). Moreover, in vitro experimental work has shown that even the weakest of electrical synapses (cc between 0.01 and 0.05) increase synchrony of subthreshold rhythms (Long et al., 2004). While weaker than other reports for juvenile TRN, homocellular and heterocellular electrical synapses measured here are not unusually weak compared with previous reports of electrical coupling in the inferior olive (Devor and Yarom, 2002) and fast spiking cortical neurons (Deans et al., 2001). In the context of a barrage of synaptic input in vivo, we would expect the somatic voltage changes induced by electrical synapses to be reduced, due to the lower input resistance. However, electrical synapses become increasingly relevant during barrages of synaptic inputs because electrical synapses transmit voltage difference, which is how electrical synapses act to improve signal-to-noise ratios and detect coincident inputs to paired neurons (DeVries et al., 2002; Dunn et al., 2006; Medvedev, 2009). Further, many TRN electrical synapses are likely located on dendrites, as they couple TRN neurons with spatially separated somas (Landisman et al., 2002; Lee et al., 2014); thus the voltage changes and coupling measured between somas are smaller than the effective ones locally at dendrites, where the electrical synapses are computationally relevant. Because TRN dendrites are endowed with active T-type calcium channels (Crandall et al., 2010), the local effect of electrical synapses on voltage at dendritic contacts are expected to be particularly important in the TRN. For heterotypic electrical synapses, each side of a coupled dendrite may receive distinct inputs or activation from the different types of thalamocortical channels. Because electrical synapses are driven by voltage differences, heterotypic synapses may be especially impactful in this configuration.

One consideration for hypothesizing about the function of homotypic and heterotypic electrical synapses in the TRN is that electrical synapses are plastic. In the TRN, repeated bursting drives depression of electrical synapses (Haas et al., 2011) and tonic spiking drives long-term potentiation (Fricker et al., 2021). Activation of different mGluRs can also cause either potentiation or depression (Landisman and Connors, 2005; Wang et al., 2015). We speculate that plasticity might be implemented distinctly between hetero- and homosynaptic electrical connections. It is possible that the TRN might maintain heterotypic synapse strength during certain brain states where connection and coordination between first-order and higher-order TRN is desirable, for example, during coordinated sleep rhythms, but be decoupled during wake states. How homotypic and heterotypic coupling changes in vivo with different wake and sleep states is an exciting question to be investigated in the future.

Acceleration of spikes within bursts has been noted for TRN neurons since early days (Domich et al., 1986; Marlinski and Beloozerova, 2014), and it has been frequently used as an identifier of the TRN for in vivo recordings. Our data clearly demonstrate that only CB neurons in the core of the TRN accelerate, while SOM neurons at the edges of the TRN rarely accelerate during bursts. Thus, it is possible that SOM cells have previously been misidentified as thalamic relay cells in vivo, and results might be re-examined in that context. Certainly, alternative methods such as spike width are necessary to distinguish SOM TRN edge cells from thalamic cells in vivo.

### Limitations of this study

The use of Cre-dependent reporter mice to label genetic subtypes has limitations. Transient expression of Cre during development can cause false expression of the fluorescence in neurons that are negative for the genetic subtype (Hu et al., 2013). This problem has been observed in adult TRN with SOM-Cre mice crossed with a tdTomato reporter mice (Martinez-Garcia et al., 2020). Our results provide evidence that this issue is avoided in juvenile tissue, as GFP expression in juvenile SOM-Cre and CB-Cre TRN was comparable with expression patterns reported in adults utilizing viral injections or in situ hybridization (Fig. 1; Martinez-Garcia et al., 2020; Hoseini et al., 2021). We did observe overexpression of GFP in SOM-Cre mice by adulthood, or after P30 (data not shown), suggesting that Cre recombination from transient SOM expression may not happen early in development, but sometime between eye opening and early adulthood. While we cannot fully rule out overexpression of GFP in our reporter mice, the electrophysiological differences between CB and SOM neurons remained (Fig. 1) and were preserved in heterocellular pairs (Fig. 3), and heterocellular coupling was not a disproportionately rare event (Fig. 4). However, differences in Cre expression could underlie the observed differences in heterotypic synapse incidence between SOM and CB lines. Experiments using a doubly labeled mouse, or post hoc immunostaining of recorded heterocellular pairs, could further enhance the results shown here; we did not perform immunohistochemistry on our tissue. Underexpression of the reporter could also impact labeling of cell types in Cre animals.

Our reports of intrinsic differences are limited to the population of GFP + neurons in our data, and misexpression or lack of GFP could cause homocellular electrical synapses to appear as heterocellular electrical synapses. Further, the overlap and spread of intrinsic properties make classification or identification of any single neuron by its individual properties uncertain. It is possible, though unlikely, that all of the synapses we have identified as heterotypic are misidentified. Therefore, it is possible for future work to refute the claim that TRN cells form both heterocellular and homocellular electrical synapses. However, we find that unlikely given the current dataset; the most supported interpretation is that TRN neurons promiscuously form electrical synapses both within and across subtypes.

The heterocellular coupling observed here occurs at the borders of SOM and CB divisions, which is another distinguishing factor of this cohort of data. Due to the nature of paired recordings, all identified somas were in close proximity to each other; our work implies but cannot fully address the possibility of coupling across larger anatomical distances or sensory sectors of the TRN. Establishing complete maps of electrical synapses across TRN functional groups remains an outstanding goal. The method used here is limited to testing one possible electrical synapse between any two neurons. It is unknown how many electrical synapses, heterocellular or homocellular, a single TRN neuron forms with its neighbors. We also note some differences in incidence and strength from previous reports of coupling in the TRN of rat, which range over mean coupling coefficients between 0.03 and 0.11 and incidence between 31 and 71% (Landisman et al., 2002; Long et al., 2004). These could be attributed to possible differences in species and in experimenter experience and bias in selecting pairs to record. Further, our determination of neuronal subtype is limited by our ability to visualize GFP during live recordings.

Our results are limited to the subtypes of neurons used here: SOM and CB neurons. We did not use PV-Cre mice, as GFP-neurons were absent from previous descriptions of crossed mice (Hou et al., 2016; Thankachan et al., 2019). Two reports suggest PV neurons are a unique subset of the TRN (Clemente-Perez et al., 2017; Hoseini et al., 2021), in a departure from the previous consensus that the TRN is nearly entirely composed of PV-expressing neurons (Seto-Ohshima et al., 1989; Frassoni et al., 1991; Mitrofanis, 1992a; Hou et al., 2016; Thankachan et al., 2019; Li et al., 2020; Martinez-Garcia et al., 2020). The results presented here do not provide any insight to this discrepancy.

Our descriptions of intrinsic differences in *R*_in_ are consistent with a previous report of SOM and CB neurons (Martinez-Garcia et al., 2020), but we note that those values differ substantially from reports that distinguish the TRN as comprising overlapping sets of SOM and PV neurons (Clemente-Perez et al., 2017) and for transcriptional differences (Li et al., 2020). Our results are consistent with previous reports of spiking differences between sets of SOM and CB or SOM and PV neurons. Both PV and CB neurons have been described as composing the core of the TRN and reciprocally connecting to primary thalamic nuclei; we thus expect that our CB results herein would also apply to PV neurons.

## Synthesis

Reviewing Editor: Arvind Kumar, KTH Royal Institute of Technology Decisions are customarily a result of the Reviewing Editor and the peer reviewers coming together and discussing their recommendations until a consensus is reached. When revisions are invited, a fact-based synthesis statement explaining their decision and outlining what is needed to prepare a revision will be listed below. The following reviewer(s) agreed to reveal their identity: Magor Lörincz.

Your manuscript was reviewed by the two reviewers. Both the reviewers think that you have done a great job in conducting experiments and reporting the results. It is indeed important to know the electrical connectivity between different types of cells, especially when the two cell types may be involved in different channels. The reviewers particularly appreciated that you have laid out the limitations of the work very clearly.

But both the reviewers have raised some valid concerns that need to be addressed in a revised version. Their main concerns are :

- To what extent the results can be generalized to in vivo-like situations e.g. when Ri is small and there is background activity. Will the gap junction still have enough SNR to be called a connection?

- It seems that heterocellular couplings are rather weak and sparse. Is it sufficient to be of functional consequence?

- Could it be that the SOM-nonSOM pairs are SOM-CB pair and CB-nonCB pairs are CB-SOM pairs?

Reviewers have made some clear suggestions about what should be included in the revised version. Please refer to the details comments from the two reviewers below.

Review #1

The authors present a nice study that aims to characterize the extent of electrical coupling among neuron subtypes of the thalamic reticular nucleus (TRN). The TRN serves as an important structure at the cross-section between the cortex and the various thalamic nuclei, and the structure is primarily composed of two different inhibitory cell types: somatostatin-positive (SOM) and calbindin-positive (CB) neurons. The presence of electrical coupling between TRN neurons is well-established. However, the current study contributes to the literature by characterizing the extent of coupling between the same TRN neuron subtypes (i.e., homocellular synapses) and between different TRN neuron subtypes (i.e., heterocellular subtypes).

The authors use cell-specific GFP mouse lines to record from SOM and CB neurons of the TRN. By recording from labeled neurons, the authors are able to characterize intrinsic electrical properties of SOM and CB neurons in juvenile mice; they establish that these properties are comparable to those reported for neuron subtypes in adult mice. The authors show that SOM and CB neurons are generally distinguishable according to their intrinsic properties. The authors then measure electrical coupling coefficients between homocellular and heterocellular pairs. The authors conclude that heterocellular coupling is relatively common in the TRN, thereby providing evidence of potential crosstalk between the two primary information channels of the thalamus (i.e., first-order and higher-order channels). Providing evidence for possibility of channel crosstalk is interesting, but I would temper the conclusion that heterocellular coupling provides evidence for functional coupling between first- and higher-order thalamic channels.

Many of the authors' conclusions rely on the assumption that SOM and CB neurons of the TRN are confidently distinguishable. In this regard, I think the authors have probably done as well as can be practically done. By coupling cell-specific GFP expression with relatively distinct electrophysiological properties, the authors can very likely correctly distinguish a SOM from a CB neuron. I am not concerned about this assumption, especially since the authors address the limitations of their approach in the Discussion. Indeed, the authors do an honest job at addressing the limitations of their study - this is refreshing to see. As described below, I think the authors should also include the limitations of solely using current clamp recordings to establish coupling coefficients. That said, I think it's a solid and interesting study.

Specific and minor comments are listed below:

Specific Comments

1. I assume that the authors characterized the electrophysiological properties of SOM and CB neurons (e.g., Figs 1 and 2) by primarily/only recording from GFP-positive cells in the relevant mouse. True? Do the data presented in Fig. 2 only come from GFP+ neurons?

Also, when characterizing the electrical coupling properties between the two cell types, do the authors only use the lack of GFP expression to guide the identification of the opposing cell type, as is suggested on lines 187-88? Or did the authors also characterize the electrophysiological properties of the non-GFP recorded cell to provide further confidence that the cell conformed to properties of the opposing cell type? For example, when characterizing SOM-CB coupling in SOM-GFP mice, did the authors characterize the electrophysiological properties of the non-GFP, presumed CB neuron to confirm that its properties were consistent with the CB properties established in Fig. 2? If the authors have this data, then it may be of interest to compare the electrophysiological properties of GFP+ neurons in SOM-GFP mice to GFP- neurons in CB-GFP mice (for example) to establish that GFP expression by itself does not skew/alter the electrophysiological properties of a neuron.

2. Was the cc between cells determined at only 1 current step, or the entire family of current steps? Also, the authors should consider more accurate tests of cc. Arguably, cc measurements can be skewed when measured in current-clamp, as the extent of voltage deflection observed in current clamp might be obscured by the activation/inactivation of voltage-dependent properties, and therefore may not reflect only the contribution of the electrical synapse but also the intrinsic properties of the coupled cells. The authors may want to check out Li et al, 2023 (Front. Syst. Biol., 08 March 2023, Sec. Integrative Systems Neuroscience, Volume 3) for more guidance. That said, the authors should be commended for including many paired recordings in their study, and providing more accurate measurements of cc using voltage-clamp seems unnecessary. However, I encourage the authors to include in their Discussion that current-clamp recordings are not ideal for measuring cc.

3. Line 198. The existence of coupling was defined when cc > 0.01. Therefore, when the injected cell generates a 10mV deflection (e.g., Fig 3B), then is an electrical synapse deemed to exist if the passive cell makes a minimum deflection of 0.1mV (100uV)? Are such deflections measurable above the noise? What do the authors mean on line 198 by "based on detectable voltage deflections". Indeed, the cc of most heterocellular synapses is quite small, less than 0.05. Does relatively weak coupling among a minority of cells provide the confidence that heterocellular synapses make a significant impact on the functional coupling of first-order and higher-order thalamic channels?

4. I'm trying to understand the significance, if any, between the different coupling rates among SOM/non-SOM pairs and CB/non-CB pairs. I recognize and appreciate that the authors likely want to define cell identity using conservative estimates (i.e., the presence or absence of GFP). But isn't it likely that SOM/non-SOM pairs are really SOM/CB pairs, whereas CB/non-CB pairs are really CB/SOM pairs? And if true, then one might expect that SOM/CB coupling rates would be similar to CB/SOM coupling. However, the authors find 23 out of 34 SOM/non-SOM synapses, but only 8 out of 19 CB/non-CB synapses. Is it possible that the synapses are rectifying, primarily allowing current to flow from one cell type to the other (e.g., SOM to CB), but not in the other direction (e.g., CB to SOM)?

Minor Comments

1. Line 1. The first sentence of the abstract appears incomplete: "inhibits sensory thalamocortical relay" neurons?

2. Line 16. The conclusion that first-order and higher-order channels are functionally linked seems strong. Yes, they are electrically coupled. But how does such coupling influence the function of either first-order or higher-order channels?

3. Line 34. I am aware of this hypothesis but the description provided here is oddly written: "to focus an attentional spotlight within the sensory surround". What does "within the sensory surround" mean?

4. Line 43. It seems like the study of Cox et al, 1996 (J Comp Neurol. 1996 Mar 11;366(3):416-30.) should also be cited here.

5. Line 92. Again, the conclusion that "heterocellular electrical synapses in TRN form networks that provide a key basis for synaptic interactions between thalamocortical first-order and higher-order channels" seems strong. The assertion that the electrical synapses provide the key substrate for cross-order interactions in the thalamus is too strong. Does the mere appearance of weak electrical connections between neurons necessitate functional relevance?

6. Lines 119 & 324. The authors indicate that horizontal slices were cut. What portion of the thalamus was evaluated? I ask because the SOM GFP in Figure 1 clearly demarks TRN. But the CB GFP in Figure 1 does not. Is it expected to find so many CB+ cells in first-order relay nuclei of rodents? Moreover, how can one be sure that some of the CB+ neuron recordings do not come from outside the TRN? As the authors indicate, "genetic recombination early in development [can] blur distinctions between cell types" (Line 109).

7. Line 134. What is meant by "Bursts in CB neurons were faster"? The bursts were characterized by a higher firing frequency?

8. Line 1 41. I would limit the figure reference for this observation to 2A. It's likely that only the aficionados will appreciate the larger voltage excursion in SOM cells in response to current injection.

9. Line 297. "PV neurons are a unique"

Reviewer #2

The MS studies the properties of electrical coupling between various classes of TRN neurons. The authors do this by performing in vitro paired recordings without post-hoc anatomy on juvenile transgenic mice. The results nicely show homo- and heterosynaptic coupling in SOM-GFP and CB-GFP neuronal populations. They also show that CB and SOM populations have distinct intrinsic membrane properties, this being more a confirmation of previous work.

The main issue with the result is the lack of neuronal identification with IHC as the ectopic expression can occur. To be fair the authors discuss these limitations in the Discussion section. Generally the MS is well written, easy to follow. Recordings performed seem to be of good quality. Analysis performed is adequate, figures informative. I think that the MS addresses the hypothesis fairly, but this is not a very complex one. In another words, it is a well written MS but not a very deep one, and remains at a rather descriptive level. Another important limitation of the study (and other in vitro studies) is that given the higher Ri of the neurons in vitro and the synaptic silence the coupling can be measured. What would happen if the Ri would be in an in vivo like range and synaptic background present? Could the authors model this? If yes what are the consequences of membrane and burst firing differences in terms of the coupling?

## Author Response

Review #1

Thank you for your detailed and supportive comments.

Specific Comments

1. I assume that the authors characterized the electrophysiological properties of SOM and CB neurons (e.g., Figs 1 and 2) by primarily/only recording from GFP-positive cells in the relevant mouse. True? Do the data presented in Fig. 2 only come from GFP+ neurons?

Yes, that is correct. Figure 2 only shows data from GFP+ neurons from their respective SOM or CB mice. We added the term "GFP+" to the figure legend of Figure 2 to improve clarity.

Also, when characterizing the electrical coupling properties between the two cell types, do the authors only use the lack of GFP expression to guide the identification of the opposing cell type, as is suggested on lines 187-88? Or did the authors also characterize the electrophysiological properties of the non-GFP recorded cell to provide further confidence that the cell conformed to properties of the opposing cell type? For example, when characterizing SOM-CB coupling in SOM-GFP mice, did the authors characterize the electrophysiological properties of the non-GFP, presumed CB neuron to confirm that its properties were consistent with the CB properties established in Fig. 2? If the authors have this data, then it may be of interest to compare the electrophysiological properties of GFP+ neurons in SOM-GFP mice to GFP- neurons in CB-GFP mice (for example) to establish that GFP expression by itself does not skew/alter the electrophysiological properties of a neuron.

For the purposes of identifying possible heterotypic synapses, we relied on the binary GFP indicator to identify Cre+ neurons only. We do provide Rin values for both cells for both homotypic and heterotypic pairs in Figure 3.

In theory, one could build a classifier based on a combination of ephys properties to speculate identity for each non-GFP neuron individually, and quantify confidence in prediction. However, this is beyond the scope and dataset of the present paper, and would ideally also use some form of scRNA-seq, for instance, to increase confidence in the range of ephys properties tied to neuron identity.

We did look at ephys properties for our 53 non-GFP neurons as a set. Their properties were generally well within the ranges of properties of the GFP+ neurons (eg Rin close ~200 MOhm, bursts with peak frequency between 200 and 400 Hz); there was no indication that GFP expression altered ephys properties.

Because the ephys properties of GFP- neurons are not definitive for specific identity; we added text at lines 305-308 to discuss this point. For this reason, we chose not to display or discuss their results, feeling that they would ultimately be a distractor.

2. Was the cc between cells determined at only 1 current step, or the entire family of current steps? Also, the authors should consider more accurate tests of cc. Arguably, cc measurements can be skewed when measured in current-clamp, as the extent of voltage deflection observed in current clamp might be obscured by the activation/inactivation of voltage-dependent properties, and therefore may not reflect only the contribution of the electrical synapse but also the intrinsic properties of the coupled cells. The authors may want to check out Li et al, 2023 (Front. Syst. Biol., 08 March 2023, Sec. Integrative Systems Neuroscience, Volume 3) for more guidance. That said, the authors should be commended for including many paired recordings in their study, and providing more accurate measurements of cc using voltage-clamp seems unnecessary. However, I encourage the authors to include in their Discussion that current-clamp recordings are not ideal for measuring cc.

Thank you for this comment. We determined cc using repeated (10x) -100 pA current steps, in order to average responses over presentations. This paradigm has been the standard for many GJ labs for years, despite its limitations. We are aware of the influence of voltage-activated conductances on GJ measurements (one of our earlier papers described effects of activating the persistent sodium current on apparent coupling; depolarization to -50 mV can change cc by 2-3x). Thus, we took care to keep neurons at -70 mV and to use only -100 pA of input current to measure coupling, which in theory should only minimally activate conductances around rest while still allowing enough voltage fluctuation in order to measure cc. To more fully address this valuable point, we have added text to the Methods (lines 390-398) to more fully describe our cc measurements and to note these considerations, including the Li work.

3. Line 198. The existence of coupling was defined when cc > 0.01. Therefore, when the injected cell generates a 10mV deflection (e.g., Fig 3B), then is an electrical synapse deemed to exist if the passive cell makes a minimum deflection of 0.1mV (100uV)? Are such deflections measurable above the noise? What do the authors mean on line 198 by "based on detectable voltage deflections". Indeed, the cc of most heterocellular synapses is quite small, less than 0.05. Does relatively weak coupling among a minority of cells provide the confidence that heterocellular synapses make a significant impact on the functional coupling of first-order and higher-order thalamic channels?

Here is the measurement of our smallest-cc heterotypic CB pair (as discussed above, traces shown are the average of 10 repetitions of hyperpolarizing current stimulus to one cell, with responses in both cells shown).

Here is the magnified view of the voltage deflections in the "receiving" neuron, with vertical lines to mark when the current pulse was turned on and off into the "active" neuron.

In our hands and eyes, we consider this clearly "detectable", coupled and above the noise floor.

To reply to the second point: The capacity for heterotypic electrical synapses to affect TRN networks at the reported strength between somas is a point worthy of discussion. We added relevant text to the discussion in lines 254-275.

4. I'm trying to understand the significance, if any, between the different coupling rates among SOM/non-SOM pairs and CB/non-CB pairs. I recognize and appreciate that the authors likely want to define cell identity using conservative estimates (i.e., the presence or absence of GFP). But isn't it likely that SOM/non-SOM pairs are really SOM/CB pairs, whereas CB/non-CB pairs are really CB/SOM pairs? And if true, then one might expect that SOM/CB coupling rates would be similar to CB/SOM coupling. However, the authors find 23 out of 34 SOM/non-SOM synapses, but only 8 out of 19 CB/non-CB synapses. Is it possible that the synapses are rectifying, primarily allowing current to flow from one cell type to the other (e.g., SOM to CB), but not in the other direction (e.g., CB to SOM)?

We do speculate that non-SOM/SOM and CB/non-CB pairs may both be SOM/CB pairs, but as the reviewer understands, we feel the need to be conservative in our interpretations. So yes, we agree that we might expect similar incidences in the cross-type coupling

A Fisher's test indicates that the difference in frequencies for CB/non-CB and SOM-nonSOM was not different (p = 0.09). We added a sentence to indicate that result in lines 208-210.

While synaptic rectification is an interesting possibility that could in theory result in apparent differences in incidence, and could arise from the different intrinsic properties of CB and SOM neurons, we looked for coupling asymmetry in our data, and found no significant differences.

Minor Comments

1. Line 1. The first sentence of the abstract appears incomplete: "inhibits sensory thalamocortical relay" neurons?

Thank you, the word neurons has been added.

2. Line 16. The conclusion that first-order and higher-order channels are functionally linked seems strong. Yes, they are electrically coupled. But how does such coupling influence the function of either first-order or higher-order channels?

Please see our reply to point number 5 below, which addresses this issue.

3. Line 34. I am aware of this hypothesis but the description provided here is oddly written: "to focus an attentional spotlight within the sensory surround". What does "within the sensory surround" mean?

We added the phrase "on specific items" to this phrase, which hopefully clarifies our intent.

4. Line 43. It seems like the study of Cox et al, 1996 (J Comp Neurol. 1996 Mar 11;366(3):416-30.) should also be cited here.

Thank you, we now cite Cox et al, 1996 there.

5. Line 92. Again, the conclusion that "heterocellular electrical synapses in TRN form networks that provide a key basis for synaptic interactions between thalamocortical first-order and higher-order channels" seems strong. The assertion that the electrical synapses provide the key substrate for cross-order interactions in the thalamus is too strong. Does the mere appearance of weak electrical connections between neurons necessitate functional relevance?

We removed the word "key" from this sentence, as we cannot make concrete conclusions about function from our data. Our added text in lines 254-275. also addresses this point.

6. Lines 119 & 324. The authors indicate that horizontal slices were cut. What portion of the thalamus was evaluated? I ask because the SOM GFP in Figure 1 clearly demarks TRN. But the CB GFP in Figure 1 does not. Is it expected to find so many CB+ cells in first-order relay nuclei of rodents? Moreover, how can one be sure that some of the CB+ neuron recordings do not come from outside the TRN? As the authors indicate, "genetic recombination early in development [can] blur distinctions between cell types" (Line 109).

We appreciate the concern. We recorded across all sensory sectors in TRN. Prior to and after recording, we visually evaluated the location of electrodes under 4x IR DIC. An example of such visualization can be seen in extended figure 1A. Under the IR light that we used to target neurons, TRN fortunately forms a fairly distinct border with relay thalamus even in high magnification, and so we are very sure that all of our recordings are truly TRN.

Regarding whether one can expect CB expression in thalamus, the study by Martinez-Garcia et al. (2020) that tied CB28KD-expression to a first-order thalamic channel shows images with CB-expression in VB thalamus. Their methods included fluorescent in situ hybridization, immunostaining, and one CB-Cre mouse injected with Cre-dependent EYFP. The viral injection was an attempt to avoid hitting VB thalamus, but the image appears to show expression in that area.

An older mRNA in-situ study (Frants and Tobin, 1994, Journal of Neuroscience Research 37:287-302) shows CB28KD expression in VB thalamus. However, it is worth noting that they oddly do not see expression in TRN.

The Allen brain atlas also shows CB expression across thalamic areas. In sum, we did not expect CB expression to demarcate TRN, and did not use it for that purpose.

7. Line 134. What is meant by "Bursts in CB neurons were faster"? The bursts were characterized by a higher firing frequency?

Yes. The text there has been changed to "Spikes within a burst from CB neurons (Fig. 1D) were faster and more numerous."

8. Line 1 41. I would limit the figure reference for this observation to 2A. It's likely that only the aficionados will appreciate the larger voltage excursion in SOM cells in response to current injection.

Good point. We now only reference 2A.

9. Line 297. "PV neurons are a unique"

Thank you, we fixed this error.

Reviewer #2

The MS studies the properties of electrical coupling between various classes of TRN neurons. The authors do this by performing in vitro paired recordings without post-hoc anatomy on juvenile transgenic mice. The results nicely show homo- and heterosynaptic coupling in SOM-GFP and CB-GFP neuronal populations. They also show that CB and SOM populations have distinct intrinsic membrane properties, this being more a confirmation of previous work.

The main issue with the result is the lack of neuronal identification with IHC as the ectopic expression can occur. To be fair the authors discuss these limitations in the Discussion section. Generally the MS is well written, easy to follow. Recordings performed seem to be of good quality. Analysis performed is adequate, figures informative. I think that the MS addresses the hypothesis fairly, but this is not a very complex one. In another words, it is a well written MS but not a very deep one, and remains at a rather descriptive level. Another important limitation of the study (and other in vitro studies) is that given the higher Ri of the neurons in vitro and the synaptic silence the coupling can be measured. What would happen if the Ri would be in an in vivo like range and synaptic background present? Could the authors model this? If yes what are the consequences of membrane and burst firing differences in terms of the coupling?

These are great points. In vivo, we expect that the effects of electrical synapses will be amplified by interactions with active conductances, as we discussed above - the persistent sodium current, for example, amplifies coupling coefficients. In the context of lower Rin in vivo, we expect that gap junctional contributions will remain important contributors to both modulation of activity and to Rin directly. We added text in response to reviewer 1 that also addresses this point, in lines 254-275, especially locally at dendritic locations of GJs.

Our group has used computational models to better understand the contributions of electrical synapses to thalamic function, and to understand the interactions between electrical synapses and other contributors to excitability and spiking in TRN neurons. Because exploring different regimes and sources of activation is a many-dimensional problem, we must consider it beyond the scope of the current manuscript. We deeply appreciate the reviewer's interest in models that include electrical synapses, and we will continue to build such models in future work.
