# Supplementary figures and images for: Functionally Distinct Circuits Are Linked by Heterocellular Electrical Synapses in the Thalamic Reticular Nucleus
Source: eNeuro. 2024 Jan 10;11(1):ENEURO.0269-23.2023. doi: 10.1523/ENEURO.0269-23.2023 (PMC10849028; doi:10.1523/ENEURO.0269-23.2023)

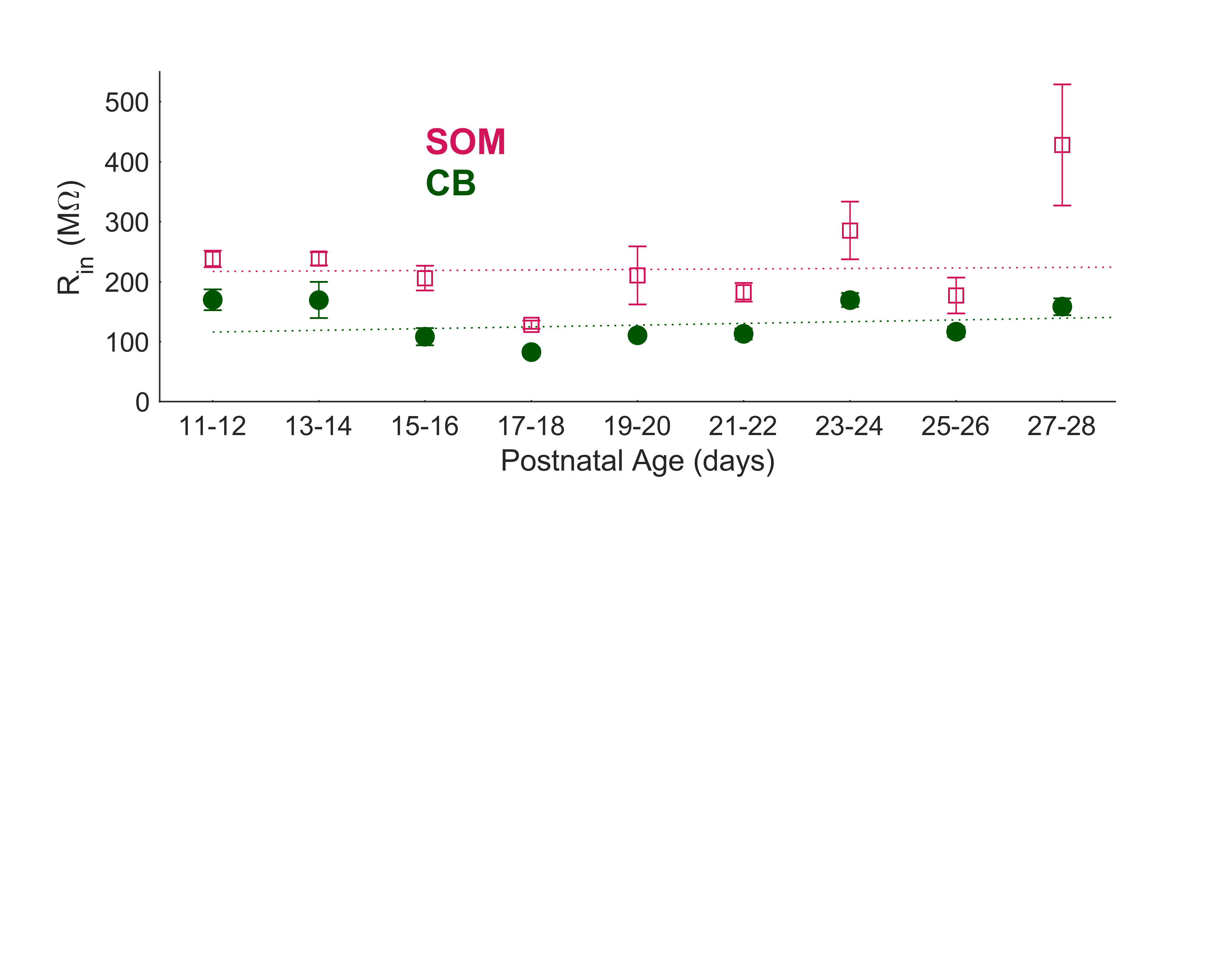

Supplement: Figure 1-1 — A. Example of within-TRN location of boundaries between expressing and non-expressing areas targeted for paired recordings Left: IR image. Right: GFP image. B. Pair targeted in ATRN. FO, first order; HO, higher order. Download Figure 1-1, TIF file. [file eneuro-11-ENEURO.0269-23.2023-s002.tif]

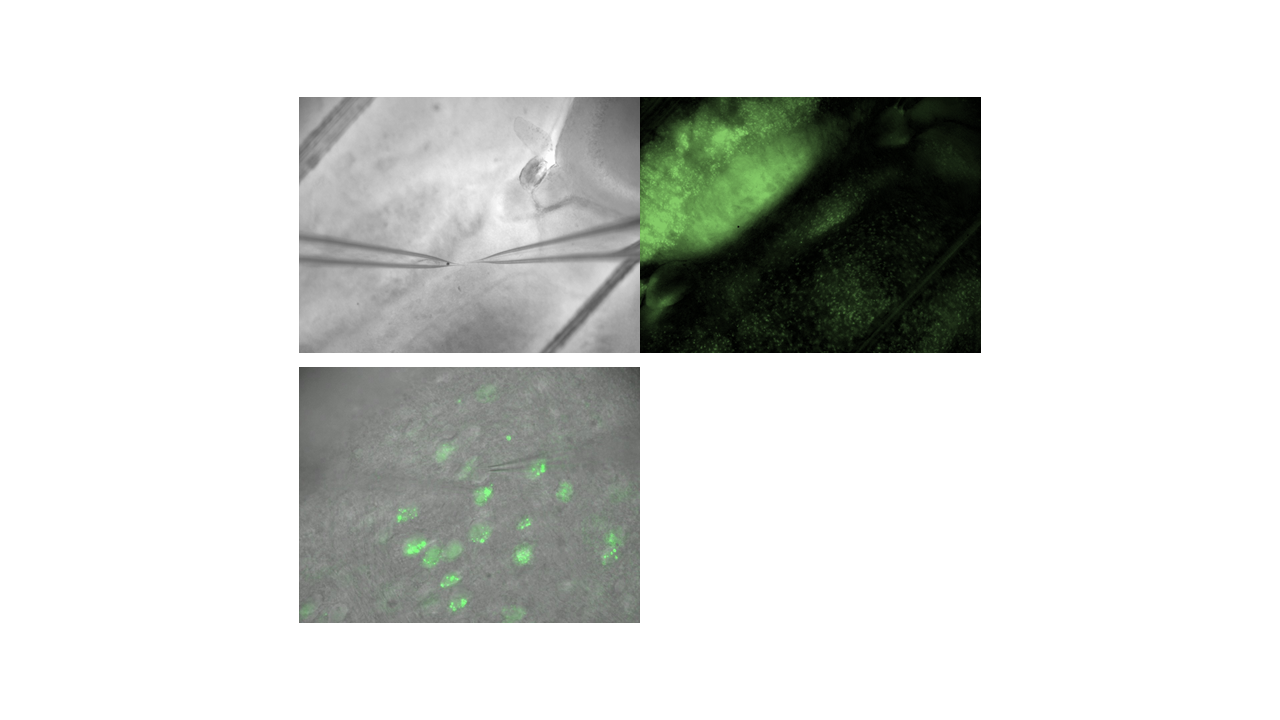

Supplement: Figure 2-1 — Input resistance (mean ± SEM) over postnatal age for the dataset used. Fits (dotted lines) are linear. Download Figure 2-1, TIF file. [file eneuro-11-ENEURO.0269-23.2023-s003.tif]

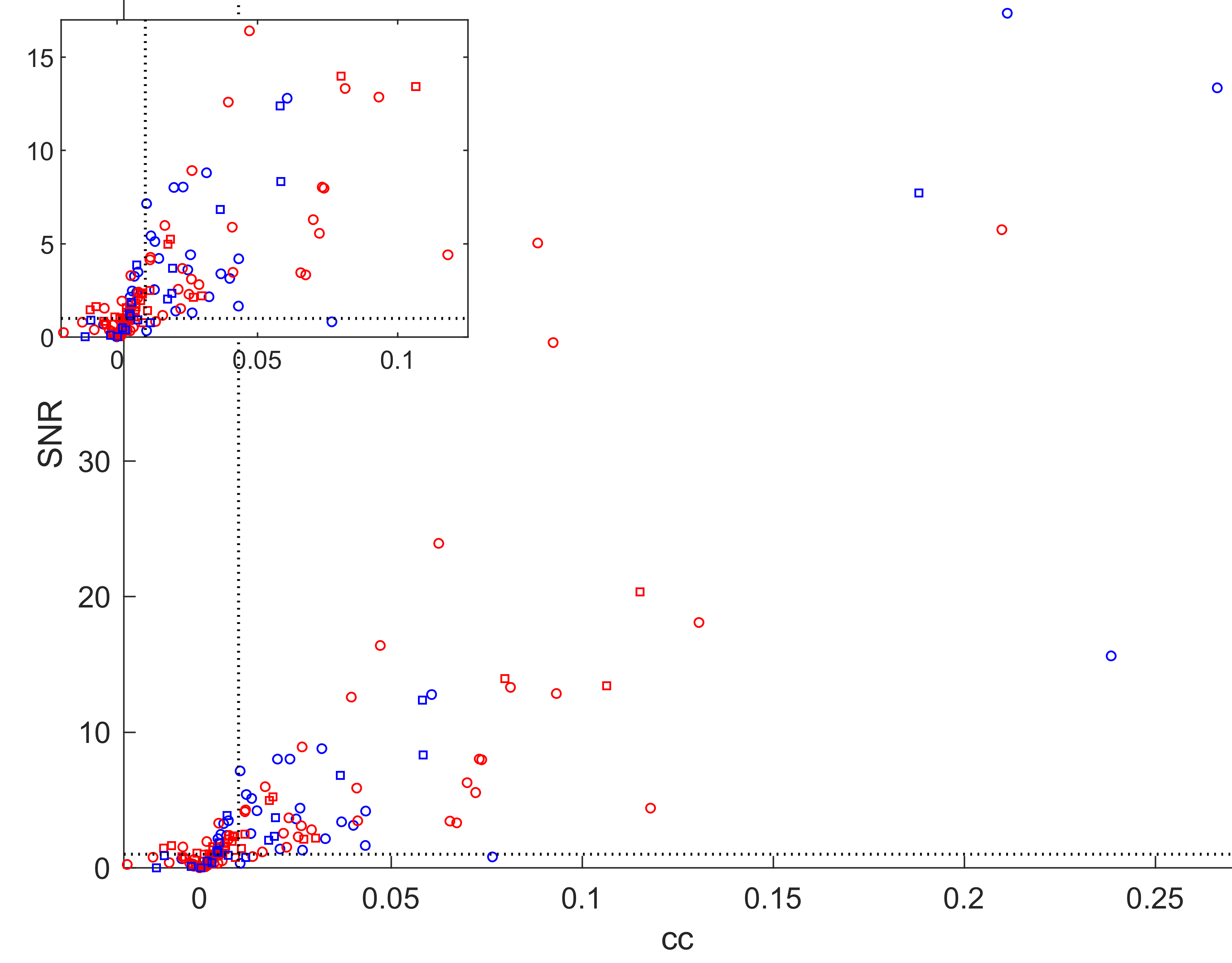

Supplement: Figure 4-1 — Signal-to-noise of coupling measurements. SNR is change in membrane voltage in the receiving neuron during repeated current steps (see Methods) divided by the standard deviation of voltage. Heterotypic pairs are blue, homotypic pairs are in red; circles represent SOM data, and squares represent CB data. Line at cc = 0.01 represents threshold used to identify electrical coupling. Download Figure 4-1, TIF file. [file eneuro-11-ENEURO.0269-23.2023-s004.tif]
